# Luciferase fragment complementation imaging in preclinical cancer studies

**DOI:** 10.18632/oncoscience.45

**Published:** 2014-06-01

**Authors:** Madryn C. Lake, Eric O. Aboagye

**Affiliations:** ^1^ Comprehensive Cancer Imaging Centre, Faculty of Medicine, Imperial College London, Hammersmith Hospital, London

**Keywords:** Luciferase, Imaging, Cancer

## Abstract

The luciferase fragment complementation assay (LFCA) enables molecular events to be non-invasively imaged in live cells *in vitro* and *in vivo* in a comparatively cheap and safe manner. It is a development of previous enzyme complementation assays in which reporter genes are split into two, individually enzymatically inactive, fragments that are able to complement one another upon interaction. This complementation can be used to externally visualize cellular activities.

In recent years, the number of studies which have used LFCAs to probe questions relevant to cancer have increased, and this review summarizes the most significant and interesting of these. In particular, it focuses on work conducted on the epidermal growth factor, nuclear and chemokine receptor families, and intracellular signaling pathways, including IP_3_, cAMP, Akt, cMyc, NRF2 and Rho GTPases. LFCAs which have been developed to image DNA methylation and detect RNA transcripts are also discussed.

## INTRODUCTION

Luciferase enzymes are widely used in the laboratory to meet a variety of experimental needs. They are employed in numerous assays for the visualization and quantification of cellular processes, including the analysis of cell fate, transcriptional activation, cellular signaling pathways and protein-protein interactions. As a preclinical optical imaging technique, bioluminescence can be seen to have a number of core strengths; the assays are comparatively cheap and safe and can be performed in a low or high-throughput fashion in a number of different contexts (e.g. in cell lysates, live cells and in living animals) relatively quickly and easily. However, the unrivalled advantages of bioluminescence are the sensitivity and capacity for imaging signal dynamics. Bioluminescence imaging (BLI) is capable of detecting very low intensity signals which can be quantified over several orders of magnitude. This super-sensitivity is derived from the absence of a background signal in unmodified cells and tissues, and distinguishes the technique from fluorescence imaging which typically suffers from a high background noise. Furthermore, because of the nature of the signal, bioluminescence lends itself to repeat imaging of the same subjects or samples. This can be particularly useful for imaging signal dynamics and for longitudinal studies. Repeat imaging of the same sample, rather than different cohorts, reduces data variability and can significantly reduce the number of subjects required for *in vivo* studies.

### Luciferase enzymes

Bioluminescent light production is dependent on luciferase enzymes, which have been isolated from a variety of sources, including bacteria, insects and a number of different marine organisms [1]. Although all of the identified luciferase enzymes are oxygenases, they are non-homologous and the ability to emit light is therefore thought to have evolved more than once [2].

Luciferase enzymes from different organisms emit distinct light spectra. Although, in general, the luciferases of pelagic and deep sea organisms emit in the blue spectrum (450-490nm), coastal marine organism luciferases emit in the green spectrum (490-520nm) and terrestrial and fresh water derived luciferases emit in the yellow-green spectrum (550-580nm) [1]. The specific wavelength emitted can also be influenced by the luciferase substrate, the enzymatic environment and presence or absence of accessory proteins [1].

**Table 1:** Characteristics of key enzymes used in luciferase complementation assays

	Firefly (Photinus pyralis)	Sea Pansy (Renilla reniformis)	Gaussia (Gaussia princeps)	Click Beetle Red (Pyrophorus plagiophtalamus)	Click Beetle Green (Cratomorphus distinctus)
Amino acids	550	311	185	542	542
Substrate	D-luciferin	Coelenterazine	Coelenterazine	D-luciferin	D-luciferin
Cofactors	02, ATP, Mg2+	02	02	02, ATP, Mg2+	02, ATP, Mg2+
Kinetics	Glow	Flash	Flash	Glow	Glow
Emission	578nm	480nm	480nm	618nm	543nm
Notable features	red shifted to 612nm at 37°C [3]		secreted	pH independent	pH independent
Key References	[12,13,26,27]	[28,79,80]	[29,60,78]	[30,31,49,58]	[30 31,81]

While luciferases have been identified in many different organisms, relatively few are commonly used in the laboratory; Table 1 lists some of the most commonly used luciferase enzymes with key references for these. The wavelength of light emitted by these enzymes ranges from blue to red, and this is an important consideration in selecting a luciferase enzyme for a specific assay. If the assay is to be used in living subjects, then a luciferase with an emission spectrum above 600nm is highly desirable. This is because light absorption by tissue elements, particularly haemoglobin and water, is greatest in the blue green spectrum and is significantly less at wavelengths above 600nm [3, 4].

Another important distinction between the different luciferases is the substrate they use. *Renilla* and *Gaussia* luciferases use coelenterazine as a substrate while the click beetle and firefly luciferases use D-luciferin. The enzymes which use coelenterazine as a substrate exhibit “flash” kinetics; maximum light production is observed within seconds of substrate addition, after which the signal rapidly declines. This is contrary to the signal emitted from the click beetle and firefly luciferase, which is relatively stable and long lasting [5, 6]. D-luciferin also has good bioavailability, making it especially useful for *in vivo* studies [6-8]. Although *in vivo* imaging with coelenterazine is possible, the substrate is relatively unstable in plasma and has an unfavorable biodistribution [6].

For the reasons mentioned above, namely the emission spectra and substrate availability, firefly and click-beetle red luciferases are considered the most suitable for *in vivo* studies.

### The luciferase fragment complementation assay

**Figure 1:**
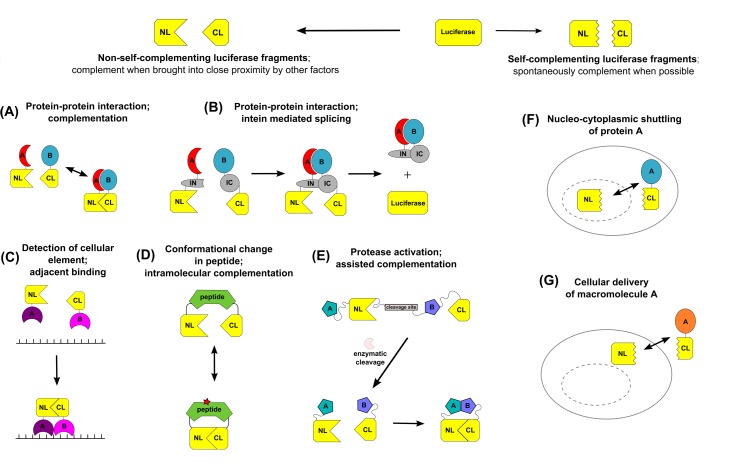
Summary of luciferase fragment complementation strategies The luciferase enzyme can be split into fragments (NL and CL), which, depending upon the specific site of cleavage, will or will not spontaneously complement. Fragments which do not spontaneously complement are able to complement when brought into close proximity. Such fragments can be used to image the interaction of two proteins of interest by either a simple complementation strategy (A) or by a split-intein (NI and CI) mediated splicing strategy (B). The complementation strategy (A) is reversible and enables interaction dynamics to be visualized. In addition to imaging protein-protein interactions, luciferase fragment complementation can also be used to image the presence of a specific cellular factor by adjacent binding (C), a conformational change in a peptide/protein (D) or protein/peptide cleavage (E). In this strategy, the luciferase fragments are fused to two self associating peptides (A and B), which are only able to bind to one another once enzymatic cleavage has occurred. Luciferase fragments which spontaneously complement have also been identified and are thought to offer the potential to image protein localization (E) and cellular macromolecule delivery (F).

The luciferase fragment complementation assay (LFCA) is a development of protein fragment complementation assays which were developed using ubiquitin, β-galactosidase and dihydrofolate reductase [9-12]. The basis of the assay is that the luciferase enzymes can be split into N-terminal and C-terminal fragments (NLuc and CLuc, respectively), which in isolation are enzymatically inactive. However, when the NLuc and CLuc fragments are brought into close proximity they are able to complement one another and luciferase enzyme activity is restored. At its simplest, this can be applied to studying protein-protein interactions by fusing the NLuc and CLuc fragments to two interacting proteins of interest. When the proteins interact, the luciferase fragments are brought into close proximity and complement, enabling the interaction to be visualized by the restoration of luciferase activity (Figure 1, A). A variation of this strategy, thought to have much potential for imaging short-lived or low-frequency interactions, entails covalently linking the NLuc and CLuc fragments upon protein-protein interaction using DnaE intein mediated splicing [12, 13]. In this strategy, interaction of the proteins of interests brings together the N and C terminal fragments of DnaE, which reconstitutes the complete intein and results in the splicing together of NLuc and CLuc to produce a full length luciferase (Figure 1, B). This produces a constitutive luciferase signal which accumulates as signaling continues. An alternative approach to restoring luciferase activity by proximity is to fuse the NLuc and CLuc fragments to two probes which are able to bind to adjacent regions (Figure 1, C). This method has been employed by different groups to image changes in the nucleic acids within a cell [14-16].

LFCA strategies have also been developed to image cellular activities that involve a protein undergoing a conformational change, which encompasses many different aspects of cellular signaling. In order to achieve this, a single fusion protein in which NLuc and CLuc fragments are cloned either side of a protein/peptide sequence, or series of protein/peptide sequences, which undergo a conformational change under specific cellular conditions is engineered. A conformational change in the protein(s) alters the proximity of the luciferase fragments and subsequently the enzymatic activity (Figure 1, D). This method has been employed to image diverse cellular processes, including the activation of protein kinases, existence of specific RNA molecules and the presence of glucose or galactose [17-19].

A further application of LFCA is the non-invasive imaging of protease activation. This has been achieved through principally similar methods; the NLuc and CLuc fragments are fused to self-associating proteins that are prevented from interacting by a peptide hindrance. When the protease of interest is activated, it cleaves the peptide and releases the fusion proteins, thereby enabling interaction and complementation (Figure 1,E). Coppola et al. imaged caspase-3 activation by fusing the NLuc and CLuc fragments of firefly luciferase to the interacting proteins peptide A and peptide B, and separating them by a caspase 3 DEVD cleavage site [20]. Shekhawat and colleagues imaged protease activation using self-associating coiled coils to assist luciferase fragment complementation. Activation of the protease of interest resulted in the cleavage and release of an autoinhibitory coiled-coil, which enabled luciferase fragment-fused complementary coils to associate and elicit restoration of luciferase activity [21].

Although the prevalent strategy in LFCAs is to use luciferase fragments which require assistance to complement, self-complementing luciferase fragments have been identified and are thought to offer the potential for studying protein localization and the delivery of macromolecules to cells (Figure 1, F and G [22]).

To date, by far the most commonly employed luciferase for LFCAs is that of the North American firefly, *Phontinus pyralis*. It is perhaps not chance that this is also one of the most widely studied luciferases. Crystal structure analysis of firefly luciferase has shown that the enzyme has a globular structure, with a large N-terminal domain and small C-terminal domain joined by a flexible hinge region [23-25]. The active site is believed to reside in the cleft between the two domains. The globular structure of firefly luciferase is thought to be particularly fortuitous for LFCAs. Empirical studies have determined that the best region to split firefly luciferase for complementation assays is within the flexible hinge region between the globular N- and C-terminal domains [26, 27]. However, no single preferred dissection site has been identified; the specific fusion protein context appears to have a significant impact on the restoration of enzymatic activity, and so different studies have found that subtly different NLuc and CLuc fragments produce the best signal:noise ratio. Although the knowledge base for the other commonly used luciferases is less comprehensive, *Renilla, Gaussia* and click beetle luciferases have all been used to image molecular events using LFCA [28-30]. By using two luciferases with different emission spectra, simultaneous imaging of more than one molecular event within a single cell has also been achieved by different groups [30-32].

### Advantages of the luciferase fragment complementation assay

As previously discussed, luciferase imaging is a highly sensitive, safe and cheap technique which can be performed numerous times on the same sample in a relatively high-throughput fashion *in vitro* and *in vivo*. In addition to these practical advantages, the LFCA has distinct benefits from the view of experimental design. These benefits principally revolve around the fact that the luciferase fragment fusion proteins are primed and ready to respond to stimuli rapidly, and are able to freely, and repeatedly, associate/dissociate as required.

In cell free systems and cellular lysates, it has been demonstrated that a plateau in luciferase fragment complementation is generally achieved within 1 or 2 minutes of ligand addition [19, 26, 33], although some studies have indicated that a plateau is reached in as little as 5 seconds [34]. From this, it has been inferred that the process of luciferase fragment complementation does not significantly impact the timeframe with which cellular processes can by visualized, i.e. the delays observed are a product of the cellular process, not the act of enzyme restoration. This potential for near real-time visualization of cellular processes is particularly advantageous given that the sample can be repeatedly imaged, thereby allowing the dynamics of a cellular response to be recorded over seconds, minutes or hours.

In addition to being able to detect the kinetics of stimulus response, LFCAs can also be used to visualize the termination of signaling because the complementation observed between luciferase fragments is reversible [29, 30, 35]. This is in contrast to fluorescent protein complementation assays, which have been shown to be irreversible due to the folding and maturation of the fluorescent β-barrel [36, 37]. Depending on the system being studied, inhibitor induced alterations in complementation between luciferase fragments has been observed within seconds or minutes, again suggesting that the timeframe of luciferase fragment dissociation does not significantly impact the visualization of cellular responses.

### Preclinical cancer studies using the LFCA

#### Cellular receptors

One of the principal areas to which LFCA has been applied is the modulation of cellular receptors. This has been achieved through a number of different strategies: receptor hetero- and homo-dimerization, receptor-effector interaction and ligand induced alterations in protein conformation. The activity of a number of different receptors has been investigated using LFCA. However, in this review, we will focus on the work conducted on 3 receptor families which are particularly significant in oncology and have attracted a sizable body of work.

### LFCA imaging of the epidermal growth factor receptor family

The epidermal growth factor receptor family, which consists of EGFR/Her1, Her2, Her3 and Her4, has been implicated in a variety of different cancer types. The receptors are tyrosine kinases which homo-or heterodimerize upon ligand stimulation. Receptor dimerization leads to activation of the intracellular kinase domain, autophosphorylation, the recruitment of adaptor proteins and the subsequent activation of intracellular signaling pathways for signal propagation. EGFR and Her2 are probably the most widely studied family members, not least because they are also most heavily implicated in tumorogenesis. EGFR is overexpressed in more than 60% of non-small cell lung carcinomas (NSCLC) and activating mutations have been detected in approximately 20% of all NSCLC patients [38, 39]. Her2 is amplified or overexpressed in 20-30% of breast cancer patients, and is associated with a more aggressive disease and worse prognosis [40]. The involvement of the EGFR family in numerous different cancers has made them the target therapeutic intervention, and as such methods to image the activity of the receptors have been investigated by several different groups.

By producing EGFR-NLuc and EGFR-Cluc fusion protein constructs, Yang and colleagues have non-invasively imaged the conformational changes that occur in EGFR after ligand stimulation [41]. Contrary to their initial expectations that EGF stimulation would increase receptor dimerization, and therefore luciferase activity, the group found that the addition of EGF produced a rapid decrease in luciferase activity followed by a recovery which lasts approximately 20 minutes. Using kinase dead EGFR mutants and the EGFR tyrosine kinase inhibitor erlotinib, the group were able to show that the decrease in luciferase activity was dependent on receptor kinase activity, but was not thought to be related to the process of tyrosine phosphorylation itself because of the timeframe of the observed changes. As such, the initial decrease in luciferase activity was attributed to conformational changes in the receptor which occur after tyrosine phosphorylation. These conformational changes have previously been demonstrated by crystallographic studies, but not in cellular systems prior to their work. Using the MAPK inhibitor U0126 and by creating EGFR-Thr^669^ mutants, it was shown that the subsequent recovery of luciferase activity was dependent on EGFR stimulated activation of the MAPK pathway.

As per the original hypothesis, the group were able to image EGFR dimerization by producing C-terminally truncated EGFR fusion proteins. This was validated by comparing the timeframe of dimerization, as observed by LFCA, with the timeframe of ^125^I-EGF binding. Using these fusion proteins the group were able to show the effect of novel small molecule inhibitors on receptor dimerization [42].

Using NLuc and CLuc labeled EGFR and Her2 fusion proteins, the same group went on to examine the dynamics of EGFR/Her2 heterodimer formation following EGF stimulation [43]. Her2 is the preferred dimerization partner of EGFR, and their dimerization is thought to have important consequences for disease. The group found that the same characteristic profile of luciferase activity was observed during hetero- and homodimer formation. Using different combinations of wild-type and kinase-dead EGFR and Her2 fusions proteins, the specific mechanics of asymmetrical kinase dimer formation were explored. The group found that the binding of EGF to EGFR stimulates EGFR to undertake a “receiver” kinase position, resulting in the EGFR kinase domain being activated prior to the Her2 kinase domain - a novel finding which had not previously been appreciated.

In addition to demonstrating the mechanics of EGFR signaling, LFCAs have also been used to the image activation and inhibition of growth factor receptor signaling. Chuan-Yuan Li's laboratory has imaged the activation of EGFR and Her2 by monitoring the association of the receptor with the downstream adaptor protein Shc [44-46]. Using this strategy, the group have imaged the *in vivo* activation of EGFR and Her2 by ionizing radiation and the activation of EGFR by hyperthermia.

Using a “intramolecular complementation” strategy, Amjad Khan and colleagues have imaged EGFR activity by producing a single fusion protein which consists of the tyrosine phosphorylation site of EPS15 and the SH2 domain of p52 cloned between the N and C terminal fragments of firefly luciferase [47]. In the absence of EGFR activity, the NLuc and CLuc fragments complement, producing a high level of luciferase activity. However, activation of EGFR results in phosphorylation of the EPS15 domain, which subsequently interacts with the SH2 domain, and alters the conformation of the fusion protein to prevent complementation between the luciferase fragments. The specificity of the changes in luciferase activity for EPS15 phosphorylation and EGFR activation were demonstrated using an EPS15 mutant and by siRNA mediated knockdown of EGFR. A particular strength of the strategy used by Amjad Khan et al. is that activation *and inhibition* of EGFR can be imaged by monitoring decreases or increases in luciferase activity. Furthermore, the system also enables visualization of EGFR activity without directly modifying the receptor itself, which could lead to unintended alterations in its biological activity. However, the reporter did demonstrate some unfortunate signal attenuation over time, which limits it potential for time-course studies.

### Nuclear receptor imaging

Nuclear receptors, which have been implicated in a number of different diseases, including cancer, comprise a large family of ligand activated transcription factors which stimulate gene expression by binding to coactivator proteins. LFCA studies of nuclear receptors have principally involved the estrogen and androgen receptors, which are primarily implicated in breast and prostate cancer, respectively.

The action of the androgen receptor (AR) has been imaged using a number of different strategies by Yoshio Umezawa's laboratory. Upon ligand binding, the AR undergoes a conformational change which results in the ligand binding domain (LBD) of the receptor interacting with the receptor N-terminus. Using a firefly luciferase intramolecular folding sensor, the group have shown that the kinetics of this conformational change differs in response to different AR ligands [48].

Using a similar click beetle red intramolecular fusion protein, the same group imaged the ligand induced association of the AR-LBD with the conserved LXXLL motif of nuclear receptor coactivators [49]. This interaction is required for transcriptional activation by the AR, and can therefore be considered a measure of the receptors genomic activity.

As an alternative method to measure AR genomic activity, Umezawa's group have also produced a pair of split *Renilla* luciferase fusion proteins to quantify the translocation of the AR from the cytoplasm to the nucleus following ligand binding [50]. Fusion proteins were produced in which the N terminus of *Renilla* luciferase and DnaE were fused to a nuclear localization sequence, and the C terminus of *Renilla* luciferase and DnaE were fused to the AR. Upon nuclear translocation of the AR construct, the N and C terminal fragments of the DnaE fragments complement and splice together the two luciferase fragments, thereby restoring the enzymatic activity. Because the restored luciferase activity depends upon a slicing event, the signal is irreversible and is cumulative. It is argued that this accumulation makes the assay more sensitive and therefore more suitable for high-throughput screening and *in vivo* imaging.

In addition to its genomic actions, the AR is also able to signal in a non-genomic fashion. Umezawa's group have investigated the ligand induced non-genomic actions of AR by imaging the association of the AR with Src [51]. Interestingly, they find that some AR ligands exhibit different specificities for stimulating the genomic and non-genomic actions of the receptor, which could have important implications in the design of novel AR therapeutic agents.

**Figure 2:**
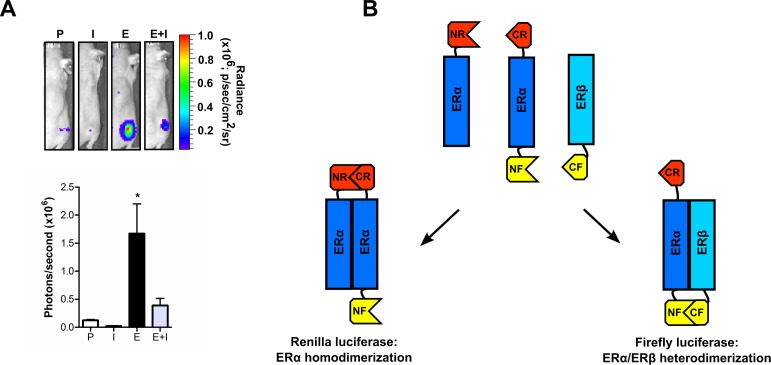
Imaging estrogen receptor biology (A) By imaging the interaction of estrogen receptor α (ERα) with the coactivator AIB1, activation of ERα by estrogen (E) and subsequent inhibition by the anti-estrogen ICI (I) can be non-invasively imaged *in vivo*. P = placebo. (B) Strategy to simultaneously image ERα/ERα and ERα/ERβ dimerization using *Renilla* (NR/CR) and firefly (NF/CF) luciferases, respectively. Figures have been adapted from [52] and [32], respectively.

Similar to the central role which the AR plays in prostate cancer, the estrogen receptor (ER) is seen as a key player in breast cancer. As a novel means of imaging the genomic actions of ER alpha (ERα), we have produced firefly luciferase fusion proteins for ERα and the nuclear receptor coactivator AIB1. AIB1 is thought to be fundamental to estrogen signaling in breast cancer, and the ERα-coactivator/AIB1 interaction is a novel target for drug development. Using ERα and AIB1 fusion proteins, we have demonstrated an estrogen dependent increase in ERα-AIB1 interaction which is modulated by anti-estrogen treatment. These alterations in luciferase activity can be observed within minutes of ligand addition and the specificity of the complementation to ERα-AIB1 interaction was confirmed using mutant ERα fusion proteins. The constructs were subsequently applied to imaging the action of ER agonists and antagonists in living subjects (Figure 2A; [52]), and are thought to offer much potential for screening novel breast cancer therapeutics, particularly those that target ERα-coactivator/AIB1 interaction.

The laboratory of Samjiv Gambhir has also investigated ER activity using the spit luciferase technique. Using an intramolecular folding sensor, in which ERα is flanked by the N and C terminal fragments of *Renilla* luciferase, ligand induced conformational changes in the receptor have been imaged [53]. By removing the C terminal F domain from the ERα fragment, they found that ligands with different pharmacologies were clearly distinguishable by the intensity of the luciferase signal produced. In the same study, the group also identified and characterized an interesting ERα mutation (G521T), which effectively abolished the binding of estrogen to ERα, but had minimal impact on the binding of other ligands [53, 54]. Using this mutant, they were able to image ligand induced modulation of the chimeric protein *in vivo,* without endogenous estrogen competing for binding with the administered compounds. It is argued that this mutant could be of significant application for the *in vivo* characterization of estrogen receptor modulators.

The Gambhir group have also investigated ERα and ERβ hetero- and homodimerization using LFCA [32]. ERβ is a nuclear receptor which shares significant sequence homology with ERα, but exhibits distinct ligand binding affinities and signaling outcomes. ERβ is not believed to be implicated in the development or progression of cancer and there is significant interest in developing and understanding the actions of ERα and ERβ specific ligands. Using LFCA, the Gambhir group showed that ERα-specific, ERβ-specific and non-specific ER ligands produce distinct patterns of ERα/ERβ homo- and heterodimerization. Using G521T mutant receptors, which are unable to bind estrogen, they were also able to show that receptor dimers can form when only one receptor is occupied by ligand.

By producing an ERα fusion protein which was N terminally tagged with the NLuc of firefly luciferase and C terminally tagged with the CLuc of *Renilla* luciferase, the group was also able to simultaneously image ERα/ERα homodimerization and ERα/ERβ heterodimerization in response to different ligands (Figure 2B). Co-transfection of this fusion protein with *Renilla-*NLuc-ERα and ERβ-firefly-CLuc fusion proteins allowed ERα/ERα homodimerization and ERα/ERβ heterodimerization to be simultaneously measured by quantifying *Renilla* and firefly luciferase activities, respectively.

### Chemokine receptor imaging

Chemokine receptors form a large family of 7-transmembrane G-protein coupled receptors (GPCR) which are activated in response to cytokine binding to mediate cell migration. Chemokine receptors have been implicated in a number of different diseases, including HIV, inflammatory diseases and a number of different human cancers.

Kathryn and Gary Luker have imaged different aspects of CXCR4 and CXCR7 biology using a variety of luciferase fragment complementation strategies. By producing NLuc and CLuc CXCR4 or CXCR7 fusion proteins they have imaged ligand induced conformational changes in CXCR4 and CXCR7 receptor homodimers [55]. Using this approach, they have shown that the timeframes of CXCR4 and CXCR7 signaling are different. However, because both receptors form dimers in the unliganded state, the changes in complementation observed using this strategy tended to be quite modest, which could limit application of this assay. As an alternative strategy to image CXCR4 and CXCR7 activation, the group produced luciferase fragment fusion protein to visualize the association of the receptors with the adapter protein β-arrestin. Employing this strategy enabled them to observe much greater increases in complementation with ligand binding and also to confirm the different time-courses of CXCR4 and CXCR7 signaling; in response to the ligand CXCL12, CXCR4-β-arrestin interaction peaks within 10 minutes and swiftly declines over the next 45 minutes whereas CXCR7-β-arrestin interaction continues to increase for up to 4 hours [56, 57]. Using the CXCR7-β-arrestin constructs, the group were also able to show that two newly identified CXCR7 antagonists, which had previously been characterized in cell free ligand binding assays, actually behave as agonists in their cellular system [57].

More recently, the group have also produced CXCR4/β-arrestin click beetle red split luciferase constructs [58]. The emission spectra from this luciferase should provide enhanced capability for *in vivo* imaging, and the group has used these constructs to demonstrate the advantages of combining cisplatin therapy with the CXCR4 inhibitor AMD3100 in mouse models of ovarian cancer.

In an innovative application of the split luciferase assay, the Luker group have also imaged the binding of the peptide ligand CXCL12 to the receptors CXCR4 and CXCR7 by producing ligand- and receptor-*Gaussia* luciferase fragment fusion proteins [59, 60]. Using this system, they were able to directly visualize the blockade of ligand binding by inhibitor treatment *in vitro* and *in vivo*, and show that inhibition of CXCL12-CXCR4 binding resulted in reduced tumor formation.

By means of another *Gaussia* luciferase complementation assay, the group has also investigated the dimerization of CXCL12, a ligand of both CXCR4 and CXCR7 [61]. By producing cells lines which express NLuc- or CLuc-fused CXCL12 the group show that CXCL12 is secreted as both a monomer and dimer, and that CXCL12 dimers can also form in the extracellular matrix. Using a range of split luciferase and non-luciferase based techniques, the group show that CXCL12 monomers and dimers activate different signaling pathways through CXCR4, and that CXCR7 preferentially binds to CXCL12 monomers rather than dimers. This could have important implications in the development of novel CXCR4 or CXCR7 antagonists.

### LFCA imaging of intracellular signaling pathways

Intracellular signaling pathways are effectors of a range of different oncogenic or tumor suppressing signals, and can be used as a means to gauge the efficacy of treatments which aim to preclude tumor formation or progression. As such, methods to image these pathways are highly desirable, and LFCAs have been produced for a number of different intracellular signaling molecules at different stages of signal transduction.

### Secondary messenger imaging

The secondary messengers IP_3_ and cAMP are produced in response to G-protein coupled receptor (GPCR) activation, and can serve as useful biomarkers for the activation or inhibition of a number of different receptors. GPCRs have been implicated in many different human diseases, including cancer, and are the targets of many established and novel anti-cancer therapies. Both IP_3_ and cAMP have been imaged by LFCA.

Using the core IP_3_-binding domain of the mouse receptor IP_3_R1 flanked by N and C terminal fragments of firefly luciferase, Ataei et al. have been able to image very rapid, transient fluxes in IP_3_ levels [62]. In cell lysates, the group observed a plateau in the luciferase signal, corresponding to a change in the conformation of the IP_3_-binding domain, 5 seconds after the addition of IP_3_. In whole cells, changes in cellular IP_3_ levels after stimulation with the peptide Bradykinin were visualized, with a peak signal 30 seconds after addition of the peptide.

**Figure 3:**
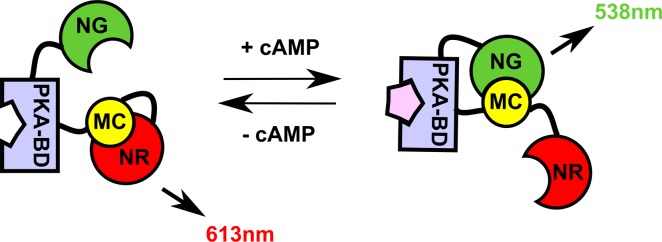
Dual wavelength ratiometric sensor for imaging cAMP levels Takeuchi and colleagues have successfully imaged cAMP levels using an engineered C terminal click beetle (CB) luciferase fragment (MC) which can complement with the N terminal fragment of CB red (NR) and green (NG) luciferases to emit red or green light, respectively. In the presence of cAMP, the intramolecular fusion protein undergoes a conformational change due to the inclusion of the cAMP binding domain of PKA (PKA-BD). Figure has been adapted from [63].

In order to image alterations in cellular cAMP levels, Takeuchi et al. produced a dual wavelength ratiometric intramolecular split luciferase sensor (Figure 3; [63]). The sensor applied a previously engineered C terminal click beetle luciferase fragment (McLuc1) which can complement with the N terminal luciferase fragments of click beetle luciferase red (NLuc-CBR) and green (NLuc-CBG) to emit light at 630nm and 525nm, respectively [31]. In this study, a single fusion protein comprising the cAMP binding domain of PKA (PKA-BD) flanked by NLuc-CBG on the amino terminal and McLuc1 and NLuc-CBR on the carboxy terminal was produced and expressed in HEK293 cells. In the absence of cAMP, the McLuc1 fragment complemented with NLuc-CBR, emitting light at 613nm, but in the presence of cAMP, an alteration in the PKA-BD conformation reduced McLuc1-NLuc-CBR complementation and promoted McLuc1-NLuc-CBG complementation, increasing emission at 538nm. By comparing the ratio of light emitted at 525nm and 630nm, the group was able to detect dose dependent increases in cAMP concentration [63]. To ensure that this ratio was not impacted by fluctuations in luciferin and ATP (while red and green filter images are sequentially captured) the group performed luciferin and ATP titrations and showed that although the individual luciferase activities were affected, the ratio of light emitted at 525nm and 630nm was largely unaffected. Using the ratiometric sensor, alterations in cellular cAMP concentrations following ISO and forskolin addition were imaged using a bioluminescence microscope with red and green long-pass filters. A modest increase in the 520/630 ratio could also be imaged *in vivo* 10 minutes after the administration of ISO. However, differential attenuation of the red and green signal could have seriously impacted the ability to observe any changes in this context, particularly given that the positive signal was in the green filter, which would have suffered greater signal attenuation than the red filter signal.

### Kinase imaging

Serine/threonine kinases play important roles in the regulation of numerous different cellular pathways, including those relevant to cancer, and have been imaged by LFCA in a number of different ways.

The activity of the key signaling molecule Akt, a serine/threonine kinases which is involved in cell proliferation and survival, has been probed in a number of different LFCA studies. Using an intramolecular folding sensor consisting of an Akt kinase substrate peptide and the phosphopeptide-binding FHA2 domain from Rad53p flanked by NLuc and CLuc fragments, Zhang and colleagues have imaged modulation of Akt activity in cells and living animals [17]. In the presence of EGF or serum, which stimulate Akt activity, a decrease in luciferase activity was observed. Conversely, an increase in luciferase activity was observed with Akt inhibitors. The specificity of the observed changes for Akt was demonstrated using mutant constructs and by immunoprecipitation of the reporter, which showed that the phosphorylation status of the reporter correlated with Akt activity. Western blotting was also used to demonstrate that the luciferase signal observed correlated with the endogenous Akt phosphorylation status. Using this LFCA, the group was able to show that the Akt inhibitors API-2 and perifosine exhibit individual pharmacodynamics *in vitro* and *in vivo*.

In a very similar study, Chan and colleagues produced an Akt kinase motif-FHA2 intramolecular fusion protein, and observed similar decreased complementation with Akt activation and increased complementation with Akt inhibition [64]. Although Chan's study used slightly different drugs and looked at reporter complementation in hours rather than minutes, the results of this study are largely similar and in agreement with the study of Zhang et al. However, an interesting addition to this study is that they use the reporter to screen 1280 compounds from the LOPAC library and identify a novel Akt inhibitor.

The phosphopeptide-binding FHA2 domain of Rad53p has also been employed to image the activity of the serine/threonine kinases GSK3β and CK1α, which have both been reported to be dysregulated in a number of different human diseases, including cancer [65]. A single fusion protein consisting of the FHA2 domain and a 20 amino acid peptide sequence derived from β-catenin, which includes substrate sequences for both kinases, flanked by NLuc and CLuc sequences was produced. In common with the previous FHA2 studies, the group found that kinase inhibition led to a dose and time dependent increase in luciferase complementation, which was shown to correlate with β-catenin phosphorylation by western blotting. Mutant reporter constructs were used to demonstrate the specificity of the signal increase for either GSK3β or CK1α phosphorylation and *in vivo* imaging demonstrated the dynamics of LiCl inhibition of GSK3β.

### Transcription factor imaging

Although the dominant method for luciferase imaging of transcription factor activation is to produce a response element-luciferase reporter construct, in which the transcription of luciferase is driven by a specific promoter element, LFCA can also be applied to imaging transcription factor activation and can be seen to have some specific advantages. Unlike transcriptional reporter assays, LFCA can image the transcription factor directly, and so the read-out is not limited to a single/ set of promoter elements. Furthermore, because the assay is primed and reversible in nature, the kinetics of activation can be visualized without relying upon cycles of transcription to produce a signal and protein turn-over to abolish a signal.

c-Myc is a transcription factor which is dysregulated in many different cancers. The frequency with which c-Myc is dysregulated in cancer suggests that it is a key regulator and, as such, has significant therapeutic potential. Fan-Minogue and colleagues have non-invasively imaged the dynamics of c-Myc by producing luciferase fragment fusion proteins to image the phosphorylation dependent association of c-Myc with GSK3β [66]. By transfecting these constructs into different cell lines, the group was able to clearly differentiate the different levels of endogenous c-Myc phosphorylation in the different cell lines, which were confirmed by western blotting. The group went on to show that the impact of MAPK inhibitors on c-Myc activation could be imaged using LFCA *in vitro* and *in vivo*, even though no alterations in tumor size could be observed.

Nrf2 is a transcription factor which is activated in response to cellular stress, particularly redox stress, to stimulate cell protective pathways. In the absence of stress signaling, inactive Nrf2 resides in the cytoplasm in association with its repressor Keap1, but upon stimulation Nrf2 is phosphorylated, dissociates from Keap1 and translocates to the nucleus. In the nucleus, Nrf2 associates with nuclear factors such as MafK and RunX2 to stimulate transcription from antioxidant response elements. Traditionally, Nrf2 has been considered a tumor suppressor, and Nrf2 activators have been developed in order to stimulate Nrf2 mediated cell protective pathways. However, new evidence suggests that Nrf2 is overactive in some cancers and can be associated with chemoresistance in these cancers, indicating that Nrf2 inhibitors could be of therapeutic use. Recently, LFCAs for Nrf2 have been developed by two independent groups to non-invasively image Nrf2 activity. Xie et al. have produced luciferase fragment fusion proteins to image the association of nuclear Nrf2 with either MafK or RunX2 [67] while Ramkumar et al. have produced a LFCA to image the dissociation of Nrf2 from Keap1 [68]. Both of these assays were developed with the specific aim of producing a robust high-throughput technique for identifying Nrf2 modulators. Although different Nrf2 activators were used in each study and the outputs were opposing (i.e. imaging association versus dissociation), the results from these studies were in accordance. While both studies were very much directed at developing a high-throughput platform, the study by Ramkumar et al. did also demonstrate the transition into *in vivo* studies using the Nrf2 activator PTS.

### Rho GTPase imaging

Rho GTPases are a subfamily of the Ras superfamily, which integrate signals from a number of different extracellular stimuli to influence cellular proliferation, gene expression and apoptosis. Rho GTPases have been shown to be dysregulated in a number of different diseases. In cancer, Rho GTPases are thought to play a role in the formation and progression of tumors, and as such, they are considered potentially valuable therapeutic targets. Leng et al. have imaged the activity of the three most widely studied Rho GTPases, CDC42, Rac1 and RhoA, through the association of the GTPases with their specific effectors: WASP, PAK and PKN, respectively [69]. The specificity of the luciferase activity for Rho GTPase-effector interaction was demonstrated by co-transfection of mismatched Rho GTPase/effector pairs and by producing mutant constructs with impaired GTPase activity or with a reduced G-protein affinity. The group was able to clearly visualize the complementation for each Rho GTPase-effector pair in cells and animals and noted that compared to similar FRET experiments, the dynamic range with LFCA was greater, suggesting that it will offer enhanced differentiation of signal modulation. The group also demonstrated that the magnitude and kinetics of activation was different for different Rho GTPase/ligand combinations.

### Imaging alterations in nucleic acid status

Nucleic acids are fundamental to cellular behavior, and alterations in nucleic acid regulation are frequently observed in human cancers. The consequences of these changes are probably most often appreciated at the level of protein expression. However, as our appreciation of epigenetics develops, so does our interest in determining the specific causes of changes in gene expression.

**Figure 4:**
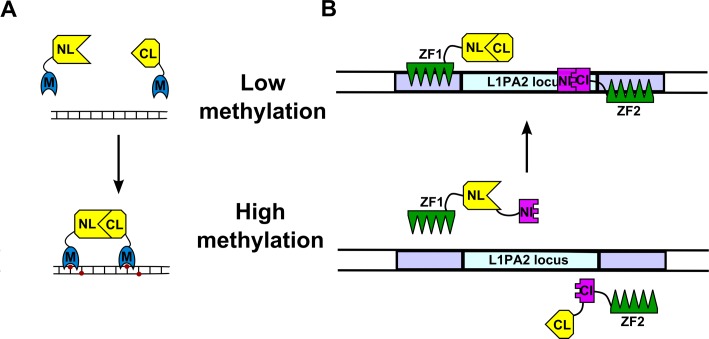
Imaging of DNA methylation (A) Badran and colleagues have imaged global DNA methylation using the CpG island binding protein MBD1 (M). In high methylation conditions, luciferase fragment fused MBD1 proteins bind to adjacent CpG islands (red dots) and complementation between luciferase fragments can occur. (B) Using artificially engineered zinc finger proteins (ZF) which recognize specific DNA sequences, Huang and colleagues have imaged methylation at the L1PA2 locus. In the absence of methylation, the DNA is more accessible and the ZF proteins can bind to the DNA, leading to complementation between the intein fragments NI and CI, and splicing together of the two luciferase fragments. Figures adapted from [14] and [15], respectively.

DNA methylation has emerged as one of the principal epigenetic mechanisms through which gene expression is dysregulated in cancer, and there is currently much interest in the development of novel epigenetic drugs to alter DNA methylation status. In order to image global DNA methylation, Badran and colleagues have developed a firefly luciferase based LFCA using the methyl CpG binding domain 1 (MBD1), which has a 110 fold preference for methylated (versus unmethylated) DNA [14]. The group produced MBD1-NLuc and MBD1-CLuc fusion proteins and demonstrate that increased luciferase activity is observed in the presence of methylated DNA, when the MBD1 fusion proteins bind to adjacent methylated CpG dinucleotides and the luciferase fragments complement (Figure 4A). The group show a dose dependent decrease in luciferase activity when purified MBD1 fusion proteins are incubated with DNA purified from cells treated with increasing concentrations of the demethylating agent decitabine. The decrease in methylation in the decitabine treated DNA was verified by methylation sensitive endonuclease digestion.

In order to assess the methylation status of specific promoters, Huang et al. used engineered polydactyl zinc finger (PZF) proteins to bind to sequences either side of the locus of interest [15]. The principle of this strategy is that methylation reduces the accessibility to the DNA, and so reduces PZF protein binding, which can consequently be used as an indirect marker of methylation. The group apply their technique to imaging the L1PA2 locus which is reportedly demethylated following azacytidine and decitabine treatment, leading to increased expression of the cMet oncogene. Two fusion proteins, each consisting of five PZF domains, a VMA intein fragment and a firefly luciferase fragment were engineered and expressed in Hela cells. Upon adjacent sequence recognition (in low methylation conditions), the intein fragments complement and splice the luciferase fragments together, resulting in restored enzymatic activity (Figure 4B). By transfecting plasmids which contain the L1PA2 target sequences into fusion protein expressing Hela cells (where the endogenous L1PA2 locus is predominantly methylated), the group were able to show an increase in luciferase activity which correlates with the presence of the unmethylated target sequence. However, sizeable increases, attributed to background intein splicing, were also observed in empty vector transfections. The group went on to demonstrate a dose dependent increase in luciferase activity in cells treated with the demethylating agents decitabine and azacitidine.

**Figure 5:**
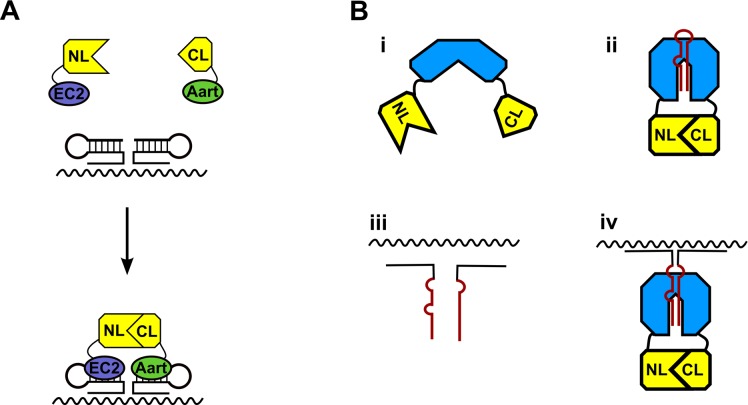
Imaging of RNA transcripts (A) Furman and colleagues have imaged the presence of specific RNA transcripts using the double stranded DNA binding proteins EC2 and Aart. Single stranded DNA probes, attached to double stranded DNA recognition sequences for EC2 and Aart, are engineered for the RNA of interest. These serve as a platform for luciferase fragment complementation. An alternative method for imaging the presence of specific RNAs has been developed by Kobatake's group. The group use the HIV-1 Rev-peptide and BIV Tat-peptide (blue polygon) which undergo a conformational change upon binding to the RNAs (depicted in red) RRE-RNA and TAR-RNA, respectively (B.i and ii). In order to detect a specific RNA of choice, the group produced split RNA probes to anneal to the RNA of interest and reform the RRE-RNA or TAR-RNA (B.iii). Once reformed, the RRE-RNA or TAR-RNA is detected by the HIV-1 Rev-peptide or BIV Tat-peptide and a change in luciferase activity is observed (B.iv). Figures adapted from [16] and [18], respectively.

RNAs, both coding and non-coding, are a major determinant of protein expression and cellular behavior, and consequently an ability to non-invasively image cellular RNAs could have a significant impact in cancer studies. Although to date limited to cell free systems, different groups have developed LFCAs to image RNAs, which could potentially be applied to the analysis of purified RNA in the future [16, 18, 70]. Perhaps the most promising of these is that of Furman and colleagues, who use DNA probes to detect specific RNA transcripts [16]. The principle of this strategy is to attach double stranded DNA recognition sequences for the high affinity zinc finger proteins EC2 and Aart to single stranded DNA probes that are complementary to adjacent regions of an RNA of interest. The double stranded EC2 and Aart binding sequences are subsequently used as a platform for luciferase fragment-fused EC2 and Aart proteins to bind, which bring the luciferase fragments into close proximity and allow the enzymatic activity to be restored (Figure 5A). Using this strategy, the group has been able to detect VEGF, HER2 and hDM2 RNA transcripts.

Eiry Kobatake's group has also attempted to image the presence of specific RNA molecules using the HIV-1 Rev-peptide and BIV Tat-peptide, which bind to the Rev-aptamers RRE-RNA and TAR-RNA, respectively [18, 70]. Binding of the peptides to their respective RNAs produces a conformational change which they have exploited to alter the proximity of flanking luciferase fragments, altering the complementation status and the subsequent luciferase activity. In cell free systems, the group has successfully produced HIV-1 Rev-peptide and BIV Tat-peptide luciferase fragment fusion proteins which elicit a specific change in luciferase activity upon exposure to their respective RNAs (Figure 5B.i and ii). The group have attempted to extend this technology to detecting a specific RNA of choice by splitting the Rev-aptamers, RRE-RNA or TAR-RNA, and fusing each half to RNA sequences which are complementary for adjacent regions of a specific RNA of choice. In the presence of the RNA, the split RNA-Rev-aptamer/RRE-RNA/TAR-RNA oligonucleotides anneal to the RNA of interest and the complete Rev-aptamer/RRE-RNA/TAR-RNA target reforms. In the presence of the reformed target, the HIV-1 Rev-peptide/BIV Tat-peptide-split luciferase probe undergoes a conformational change, which alters the complementation between the associated luciferase fragments (Figure 5B.iii and iv). This is quite a complicated system, which has principally worked in cell free systems, but the limits of detection are currently insufficient to be transferred into cellular systems.

In addition to being a cause of cancer, nucleic acids also provide an effective means of restricting cancer growth. Radiotherapy and some chemotherapies aim to induce DNA damage in order to stimulate cell death. Assess the efficacy of different treatment regimes, Li and colleagues have developed a LFCA to non-invasively image the formation of DNA double strand breaks (DSB) *in vitro* and *in vivo* [71]. The group use luciferase fragment fusion proteins to image the association of histone H2AX, which is phosphorylated in response to DSB formation, with MDC-1. Using these fusion proteins, the group demonstrated a dose dependent increase in luciferase activity following exposure to ionising radiation, which correlates with phosphorylation of the endogenous and luciferase-fused H2AX proteins. In accordance with previous findings, they observe a rapid increase in luciferase activity 30 minutes after radiation exposure, which declines within 24 hours. However, they also unexpectedly observed a second peak in H2AX-MDC-1 interaction approximately 5-10 days after radiation exposure. By measuring caspase-3 activation and PARP cleavage they show that this second wave of luciferase activity correlates with tumor cell apoptosis, which is associated with the formation of numerous DSB.

## CONCLUSIONS

Luciferase fragment complementation is a comparatively new technique which has specific strengths for use in preclinical cancer studies: it is cheap and safe; it is adaptable to low or high-throughput qualitative or quantitative research; it is readily adaptable to cell free, *in vitro* and *in vivo* studies; it is primed and reversible in nature; and it can be applied to a range of different cellular processes using various complementation or intein mediated methods.

At present, click beetle red luciferase is probably the most promising luciferase available for LFCAs. Although click beetle green has been shown to emit a stronger signal, click beetle red luciferase emits a comparatively strong signal relative to the other commonly used luciferases and its red emission spectrum is highly favorable for *in vivo* applications, particularly deep tissue othotopic imaging [3, 72]. As the optical imaging field develops, it can be anticipated that the number of studies using click beetle red luciferase will increase, and that with this the body of knowledge surrounding the luciferase will grow considerably.

Recently, click beetle red luciferase has been used in combination with click beetle green luciferase to image multiple interactions in a single cell [30, 31]. It is likely that these more complex LFCAs will increase in popularity as experimental demands and imaging systems develop. By combining LFCAs with resonance energy transfer methodologies, the quality and quantity of information extracted from a single experiment can be expected to increase even further. Dragulescu-Andrasi and colleagues have developed a red light emitting bioluminescent resonance energy transfer (BRET) technique to image deep tissue protein-protein interactions and Rebois et al. have combined luciferase and fluorescent complementation assays to image the association of 4 proteins in a single cell [73, 74]. These experiments exploit the best features of these different imaging techniques: the use of a bioluminescent protein as a excitatory light source removes the need for external illumination, resulting in a lower background signal and consequently improved sensitivity; and the use of an acceptor fluorophore increases the signal intensity, enables the emission spectrum to be red shifted and enhances spatial resolution.

To date, luciferase fragment complementation has been applied to investigating various different aspects of cellular signaling. Innovative methods have been developed to investigate ligand binding, ligand pharmacology, transmembrane and intracellular receptor signaling, signal transduction, enzyme activation, and the presence of specific macromolecules within a cell. Given the infancy of the method, many of these studies have to a greater or lesser extent contained “proof-of-principle” elements, which aim to broaden the application of the technique rather than probe novel biological questions. However, these experiments have contributed greatly to establishing the validity and potential of the assay, and as the assay becomes widespread, it can be expected that it will be applied, in combination with other techniques, to more in-depth studies of biological and oncological importance.

One of the key areas which luciferase fragment complementation holds much promise is in the field of high-through put screening. This field is particularly relevant in cancer studies; as the number of available compound libraries increase and new therapeutic targets in cancer are identified the need for high-throughput screening technologies increases. Although relatively few in number, studies are now emerging in which LFCAs have been applied to screening large numbers of compounds or the validation of compounds identified by other screening methods [42, 64, 68, 75, 76]. Recently, a *Gaussia* luciferase based assay has also been developed for the validation of protein-protein interaction networks, which has been used to confirm a human papillomavirus interaction network identified using a yeast two-hybrid screen [77, 78].

Although luciferase fragment complementation is still a relatively new technique, it is clear that it has much to contribute to the field of cancer studies. The technique can be applied to qualitative investigations of cellular signaling or quantitative studies involving large data sets equally well. This enables the assay to be applied to almost all aspects of preclinical cancer studies, and as the field develops, the innovative applications of the assay are likely to increase.
